# Immunoregulatory Cell Depletion Improves the Efficacy of Photodynamic Therapy-Generated Cancer Vaccines

**DOI:** 10.3390/ijms161126008

**Published:** 2015-11-12

**Authors:** Mladen Korbelik, Judit Banáth, Kyi Min Saw

**Affiliations:** British Columbia Cancer Agency, 675 West 10th Avenue, Vancouver, BC V5Z 1L3, Canada; jbanath@bccrc.ca (J.B.); ksaw@bccrc.ca (K.M.S.)

**Keywords:** photodynamic therapy, cancer vaccines, immunoregulatory cells, cyclophosphamide, all-*trans* retinoic acid (ATRA)

## Abstract

Photodynamic therapy (PDT)-generated cancer vaccine represents an attractive potential application of PDT, therapeutic modality destroying targeted lesions by localized photooxidative stress. Since immunoregulatory cell activity has become recognized as a major obstacle to effective cancer immunotherapy, the present study examined their participation in the therapeutic effect of PDT cancer vaccine. Following protocols from previous studies, mouse with squamous cell carcinoma SCCVII tumors were vaccinated by SCCVII cells treated by PDT and response monitored by tumor size measurement. The effects of low-dose cyclophosphamide (50 mg/kg) and all-*trans* retinoic acid (ATRA) on the numbers of Tregs and myeloid-derived suppressor cells (MDSCs) were determined by antibody staining followed by flow cytometry, while their impact on PDT vaccine therapy was evaluated by monitoring changes in tumor responses. Cyclophosphamide effectively reduced the numbers of Tregs, which became elevated following PDT vaccine treatment, and this resulted in an increase in the vaccine’s effectiveness. A similar benefit for the therapy outcome with PDT vaccine was attained by ATRA treatment. The activities of Tregs and MDSCs thus have a critical impact on therapy outcome with PDT vaccine and reducing their numbers substantially improves the vaccine’s effectiveness.

## 1. Introduction

Development of photodynamic therapy (PDT)-generated cancer vaccines [1,2] remains one of the attractive potential applications of PDT, a clinically established modality for the destruction of tumors or other lesions through localized formation of cytotoxic reactive oxygen species by light-activated drugs (photosensitizers) [3,4]. Photooxidative lesions of targeted cancer cell proteins and lipids induced by PDT elicit a complex reaction resulting in direct cell killing, damage to tumor vasculature, as well as inflammatory, acute phase and immune responses [3,4,5,6]. The induction of immune response against PDT-treated tumors is apparently caused primarily by a massive release of endoplasmic reticulum (ER) stress response proteins (acting as damage-associated molecular patterns DAMPs), delivered through the danger signaling cascade provoked by a strong oxidative stress inflicted by PDT treatment [6,7,8].

Surgically removed tumor tissue (or derived cells) exposed *ex vivo* to PDT was shown to serve as an excellent source for autologous therapeutic cancer vaccines [2,9]. The unfolded protein response (UPR) launched within the PDT-induced ER stress response promotes, when the insult remains unresolved, a specialized type of apoptosis known as immunogenic cell death (ICD) [10]. Such vaccine material, consisting of cancer cells undergoing ICD, will attract host’s sentinel efferocytes/phagocytes specialized in removal of these corpses that will process and present tumor antigenic material contained in them in an optimal way for the immune recognition and development of adaptive immune response against original tumor [2,3].

The present study was designed to address the question whether PDT-generated cancer vaccine activity is influenced by host’s immunoregulatory cells. Such cell populations were shown to become transiently elevated following treatment of several types of mouse tumors *in situ* by standard PDT [11,12]. In the same studies, it was shown that a selective depletion of regulatory T cells (Tregs) by low-dose cyclophosphamide administration improves the therapeutic efficacy of PDT in attaining cures of treated tumors. Thus, it became warranted to determine whether the potency of PDT-generated vaccines could also be improved by subduing the activity of immunoregulatory cells.

## 2. Results and Discussion

### 2.1. Results

#### 2.1.1. Low-Dose Cyclophosphamide Combined with Vaccine Treatment

**Figure 1:**
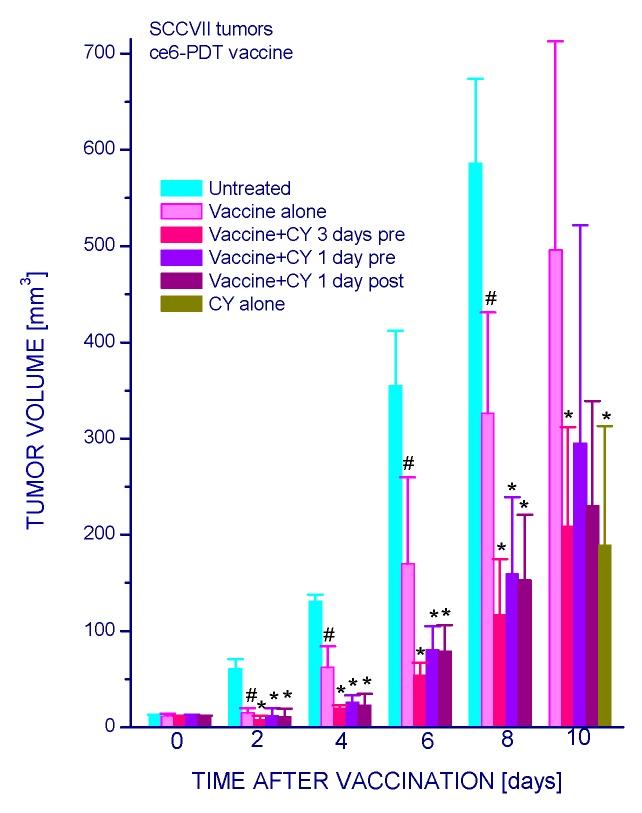
The effect of cyclophosphamide injected either before or after PDT vaccine treatment on growth of vaccinated tumors. Mice bearing subcutaneous SCCVII tumors were injected PDT vaccine cells (2 × 10^7^/mouse peritumorally) and were thereafter monitored for registering tumor growth. Cyclophosphamide (50 mg/kg) was given as a single i.p. injection at either three or one days before or one day after vaccination. Each treatment group consisted of six mice. # Statistically significant difference from untreated tumors group (*p* < 0.05); * statistically significant difference from vaccine alone group (*p* < 0.05).

**Figure 2:**
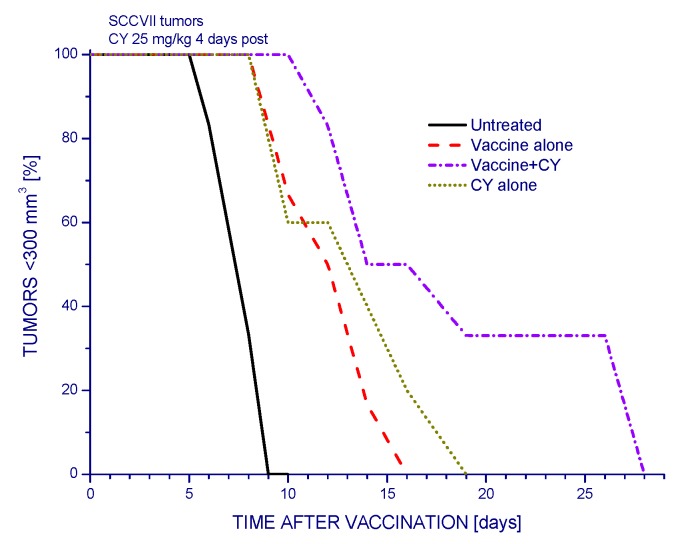
Increased efficacy of PDT vaccine when combined with cyclophosphamide treatment. Mice with SCCVII tumors were treated with PDT vaccine as described for Figure 1. Single injection of cyclophosphamide (25 mg/kg i.p.) was given four days post vaccination. The results are presented as percentages of mice with tumors smaller than 300 mm^3^ in function of time after vaccination. Each treatment group consisted of six mice. Statistically significant (*p* < 0.05) were the differences between the control untreated group response and all three treatment group responses, between vaccine alone response and vaccine plus cyclophosphamide response, and between vaccine plus cyclophosphamide and cyclophosphamide alone responses.

Low-dose cyclophosphamide has a pronounced effect on the therapeutic efficacy of PDT vaccines. The growth of SCCVII tumors that was significantly delayed following the administration of PDT vaccine was further retarded by injecting the host mice with cyclophosphamide (CY) (50 mg/kg i.p.), and this effect was similarly pronounced with giving the drug at either three or one days before vaccination, or one day after vaccination (Figure 1). However, injecting CY alone at the same dose produced a similar tumor growth slow-down as in combination with PDT vaccine. In order to demonstrate a clear benefit of CY on the impact of PDT vaccine, this drug was, in the following experiments, used at 25 mg/kg and injected four days post-vaccination (when control tumors became almost ten-fold greater than at the time of vaccination). The results, presented as Kaplan–Meier survival plots with tumor size of 300 mm^3^ as survival cutoff, show that the survival benefit attained by PDT vaccine alone was significantly extended by the adjuvant CY treatment (Figure 2). This benefit was superior to the gains reached with either PDT alone or CY alone treatments.

#### 2.1.2. Single and Double Cyclophosphamide Treatment

**Figure 3:**
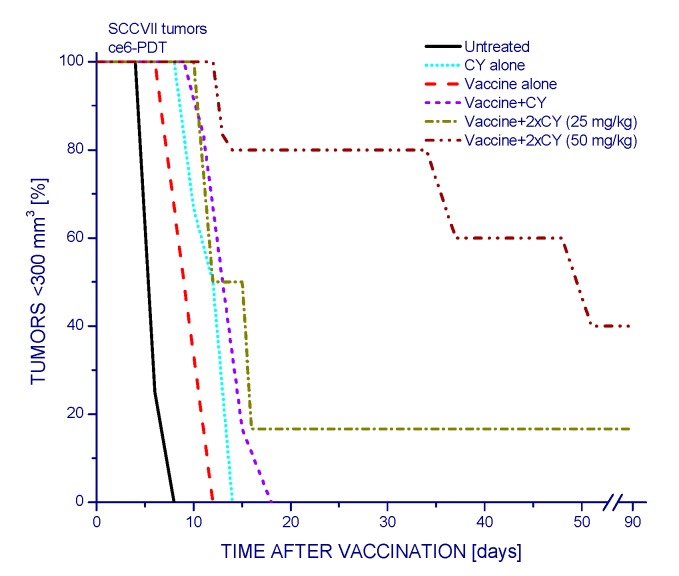
The impact of twice injected cyclophosphamide on the efficacy of PDT vaccine. Cyclophosphamide (25 or 50 mg/kg i.p.) was injected at two days before and four days post vaccination performed as described for Figure 1, and the same without vaccination was done for “CY alone” group; the higher dose single treatment (“Vaccine+CY group”) was done at two days pre vaccination. The results are presented as percentages of mice with tumors smaller than 300 mm^3^ in function of time after vaccination; mice surviving at 90 days post therapy are by convention considered cured. Each treatment group consisted of six mice. Statistically significant (*p* < 0.05) were the differences between the control untreated group response and all five treatment group responses, between vaccine alone response and all three vaccine plus cyclophosphamide groups’ responses.

The adjuvant effect of CY when combined with PDT vaccines can be further optimized by extending it from single to double treatment. The results, again presented as Kaplan–Meier plots, reveal that with 25 mg/kg CY treatments given two days before and four days after vaccination there were tumor cures in 16% of mice (no signs of tumor regrowth for over 90 days post therapy) (Figure 3). Increasing the dose of CY treatment to 50 mg/kg further improved the therapy outcome by attaining 40% tumor cures. The effects of PDT vaccine alone and CY alone were similar to those obtained in previous experiments (Figure 2).

#### 2.1.3. PDT Vaccine and Tregs

**Figure 4:**
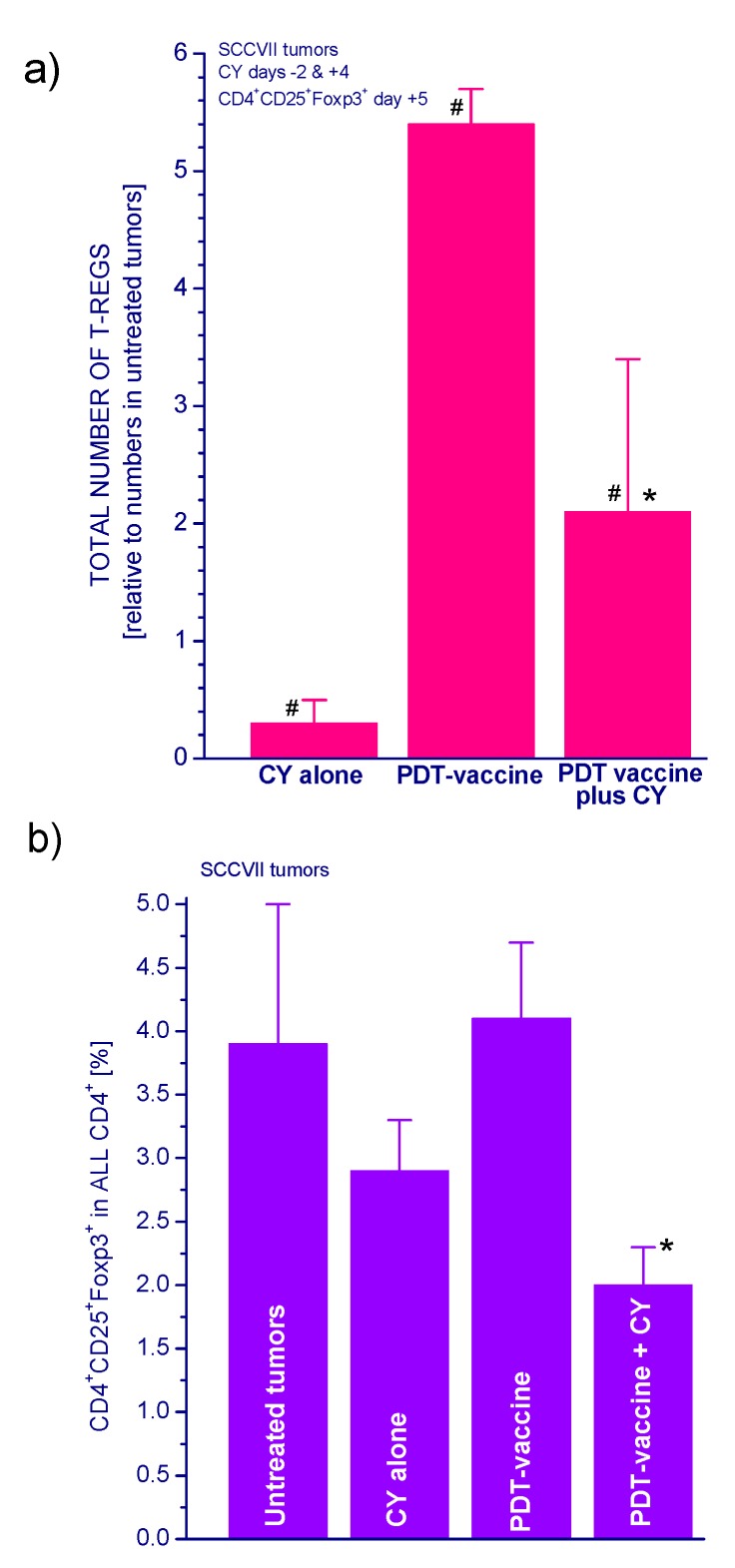
Treg numbers in untreated and treated SCCVII tumors and the effect of cyclophosphamide. Mice with SCCVII tumors were treated with PDT vaccine as described for Figure 1 and received two cyclophosphamide injections (50 mg/kg) as described for Figure 3. Mice were sacrificed five days post vaccination and cell suspensions from their tumors were stained with fluorophore-conjugated antibodies to identify Tregs as CD4^+^CD25^+^Foxp3^+^ cells using flow cytometry. The data are presented (**a**) as relative values (compared to untreated tumor) of total number of Tregs per tumor and (**b**) as percent of Tregs in helper T cell population. Each treatment group consisted of four mice. # Statistically significant difference from untreated tumors group (*p* < 0.05); * statistically significant difference from vaccine alone group (*p* < 0.05).

**Figure 5:**
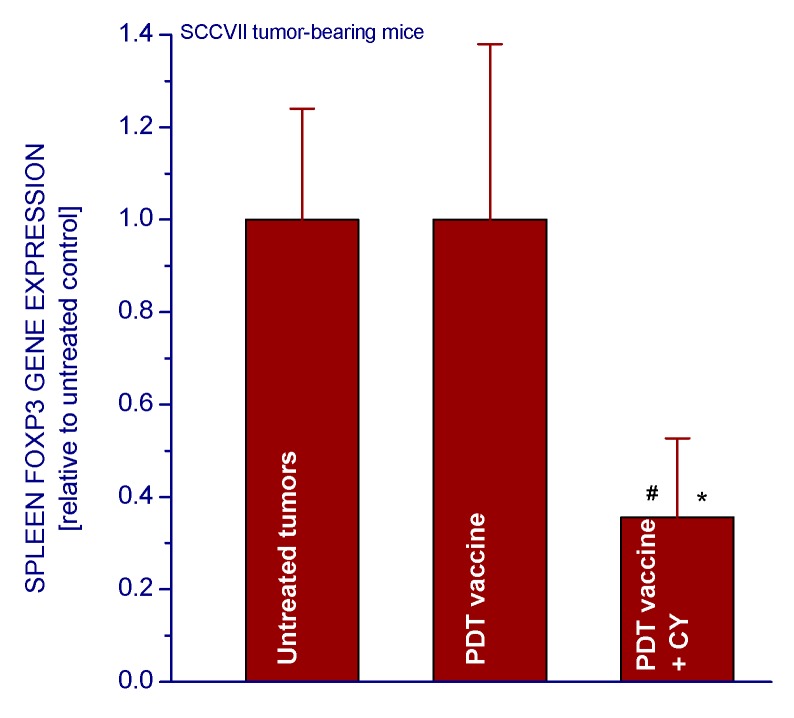
The effect of cyclophosphamide treatment on the expression of Foxp3 gene in the spleens of SCCVII tumor-bearing mice that received PDT vaccine. The mice were sacrificed three days after vaccination (as in Figure 1) followed by cyclophosphamide injection (50 mg/kg i.p.) two days later. Their spleens were collected for Foxp3 gene expression analysis based on quantitative RT-PCR. The results, GAPDH-normalized mouse Foxp3 gene expression, are presented relative to corresponding values in spleens of untreated controls. Treatment groups consisted of four mice. # Statistically significant difference from untreated tumors group (*p* < 0.05); * statistically significant difference from vaccine alone group (*p* < 0.05).

To examine whether any changes in Treg populations were induced by the investigated treatments, the tumors were collected from sacrificed mice one day after the second CY treatment (five days post vaccination) and derived cell suspensions stained to identify CD4^+^CD25^+^Foxp3^+^ cells using flow cytometry. The untreated control tumors contained on average close to one thousand Tregs, and around 5.4-fold higher numbers in PDT vaccine-treated tumors (Figure 4a). The CY treatments proved effective in reducing Treg levels by over 60%. The percentages of Tregs in helper T-cell populations of PDT vaccine-treated tumors were also significantly reduced by CY treatment (Figure 4b). The activity of Treg, which are also found in the spleens of host mice, can be monitored by the expression of Foxp3 gene [13]. In accordance with the cell number analysis (Figure 4), the CY treatment reduced the spleen Foxp3 gene expression in mice bearing PDT vaccine treated tumors by over 60% (Figure 5).

#### 2.1.4. PDT Vaccine and MDSCs

**Figure 6:**
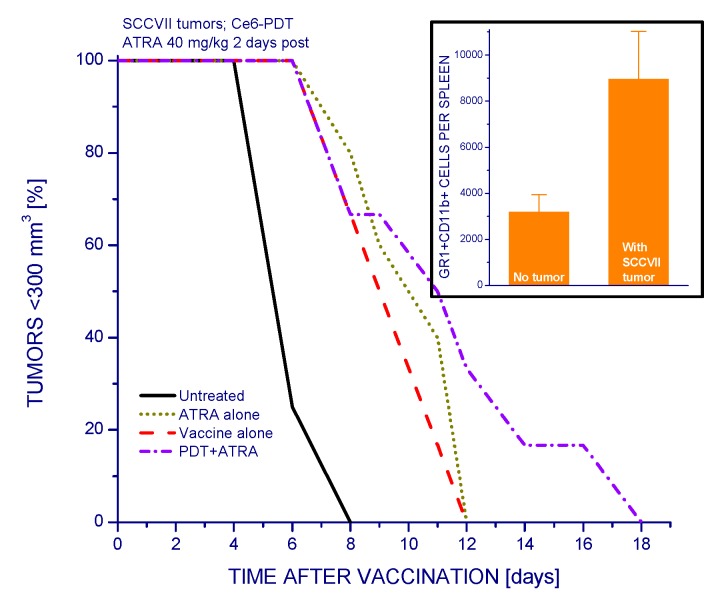
The impact of single ATRA treatment on the efficacy of PDT vaccine. Mice with SCCVII tumors were treated with PDT vaccine as described for Figure 1. The injection of ATRA (40 mg/kg i.p.) was given two days after vaccination. The results are presented as percentages of mice with tumors smaller than 300 mm^3^ in function of time after vaccination. Each treatment group consisted of six mice. Statistically significant (*p* < 0.05) were the differences between the control untreated group response and all three treatment groups responses, as well as between vaccine alone and vaccine plus ATRA groups responses. The numbers of MDSCs (GR1^+^CD11b^+^ cells) per spleen in mice with and without SCCVII tumors are shown in the insert. The difference between the two groups in the insert was statistically significant (*p* < 0.05).

Growth of SCCVII tumors prompts also an elevated activity of MDSCs in the host mice, as shown by the rise in GR1^+^CD11b^+^ cells in their spleens (Figure 6, insert). Thus it seemed warranted to test the effect of a MDSCs-modulating agent ATRA on the efficacy of PDT vaccines. As seen with survival plots, tumor growth retardation attained by PDT vaccine treatment (and in a similar fashion also by ATRA alone) was significantly extended with adjuvant ATRA administration given as a single i.p. injection two days post vaccination (Figure 6).

### 2.2. Discussion

Strength and duration of antitumor immune response can be actively attenuated by negative regulatory immune cell populations [14]. It has become increasingly clear that the activity of these immunoregulatory cells physiologically engaged in the maintenance of immunological self-tolerance is a major barrier to effective tumor immunotherapy [14,15,16]. Tumors can recruit and activate suppressive or regulatory cells from both adaptive and innate arms of the immune system. The two major populations are regulatory T cells (Tregs) [17,18] and myeloid-derived suppressor cells (MDSCs) [19]. The present work demonstrates that both these immunoregulatory populations can influence therapy outcome with PDT-generated cancer vaccines. It is shown that growth of SCCVII tumors instigates a rise in the levels of Tregs and MDSCs in the host mice. Moreover, a further increase in Treg numbers was found to be provoked after PDT vaccine treatment (Figure 4a).

The work of Hamblin’s group [12] has demonstrated that PDT of mouse tumors *in situ* leads to an increase in Treg levels in both spleen and lymph nodes during the first few days post treatment. They have also shown that reducing the numbers of Tregs in mice bearing PDT-treated tumors by adjuvant low-dose cyclophosphamide treatment (50 mg/kg) potentiated PDT-mediated immunity leading to the development of strong immune memory and improved tumor cure-rates [11,12]. Low-dose cyclophosphamide treatment is widely used as an immunotherapy adjuvant known for its ability to selectively inactivate Tregs in human cancer patients and mouse tumor models, although its enhancement of immune response against tumors may result from multifaceted properties including also the induction of “cytokine storm”, activation of homeostatic lymphoid proliferation, affecting antigen presentation, and antiangiogenic effects [20]. The results of present study demonstrate that the low-dose cyclophosphamide treatment reduces Treg levels in PDT vaccine-treated mice, which is manifested also as a decrease in the expression of forkhead transcription factor Foxp3 gene in these tumors. This transcription factor is constitutively activated in Tregs and serves as their specific marker [13,21]. The results further show that a single cyclophosphamide injection delivered three days before or four days after PDT vaccine, even when lowered to 25 mg/kg, significantly improves the vaccine’s therapeutic efficacy. Although a 50 mg/kg dose of cyclophosphamide alone is known to exert a strong inhibitory effect on mouse tumors [11,12] (Figure 1 and Figure 2), it cannot replace therapy such as PDT vaccine because by itself it is not capable of inducing antitumor immune response that enables a long-term tumor control. Furthermore, at adequate conditions, cyclophosphamide can be demonstrated to attain benefits when combined with PDT vaccine that markedly exceed the gains found with either of these agents alone (Figure 2). Moreover, injecting cyclophosphamide two times, at two days before and four days after vaccination has even greater therapeutic benefit manifested as permanent tumor cures (Figure 3). This is in accordance with the fact that the effect of cyclophosphamide is transient and Treg numbers largely recover in 5–7 days [12]; hence multiple (known also as metronomic) low-dose cyclophosphamide treatment extends the duration of Treg depletion and activity impairment [20].

Potential side-effects of low-dose cyclophosphamide are greatly reduced compared to use of it at high doses. Particularly important is lowered risk of its carcinogenic action [22]. Low-dose cyclophosphamide treatment renders mice neutropenic, but this effect is only short term.

The present study also reveals that the other physiologically relevant immunoregulatory population, MDSCs, is also involved in growth of SCCVII tumors and influences the response of these tumors to PDT vaccines. This is evidenced by the beneficial effect of ATRA (Figure 6), which reduces the MDSCs levels in carcinoma patients and tumor-bearing mice by causing their differentiation into mature myeloid cells [23,24]. It can be expected that even greater therapeutic effect can be attained by several ATRA treatments during the period before and after PDT vaccination since this agent is usually used in multiple day release/treatment regimens [23].

In conclusion, the present study uncovers the involvement of immunoregulatory cell populations Tregs and MDSCs in the events preceding and following PDT cancer vaccine treatment that have critical impact on therapy outcome. The results suggest that the therapeutic effectiveness of PDT cancer vaccines can be substantially improved by concomitant obstruction of the activity of immunoregulatory populations to allow more potent and durable tumor surveillance by the vaccine-induced antitumor immune response. This recognition is in correspondence with the growing realization that tumor-induced immune suppression is a principal obstacle to effective cancer immunotherapy, and that it needs to be addressed by establishing protocols for dampening immunoregulatory cell activity in patients under immunotherapy regimens [14].

## 3. Experimental Section

### 3.1. Tumor Model

**Figure 7:**
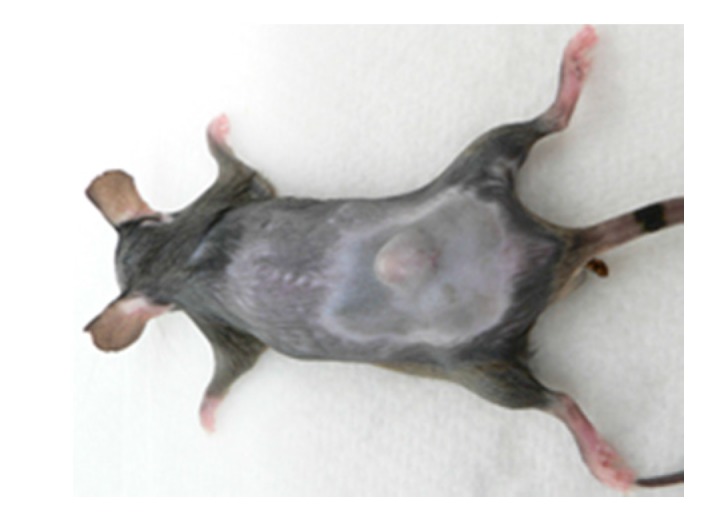
Mouse with subcutaneous SCCVII tumor growing in lower dorsum.

Squamous cell carcinoma SCCVII, an established mouse head and neck cancer model of spontaneous origin was chosen because of its limited immunogenicity [25]. Maintaining SCCVII cells in culture was done using Alpha MEM medium supplemented with 10% fetal bovine serum (Life Technologies, Burlington, ON, Canada). Subcutaneous SCCVII tumors were grown in syngeneic C3H/HeN mice by inoculating 1 × 10^6^ SCCVII cells subcutaneously into the lower dorsum (Figure 7). The experimental protocols involving mice were in compliance with the Animal Care Committee of the University of British Columbia.

### 3.2. PDT Vaccine Preparation and Treatment

**Figure 8:**
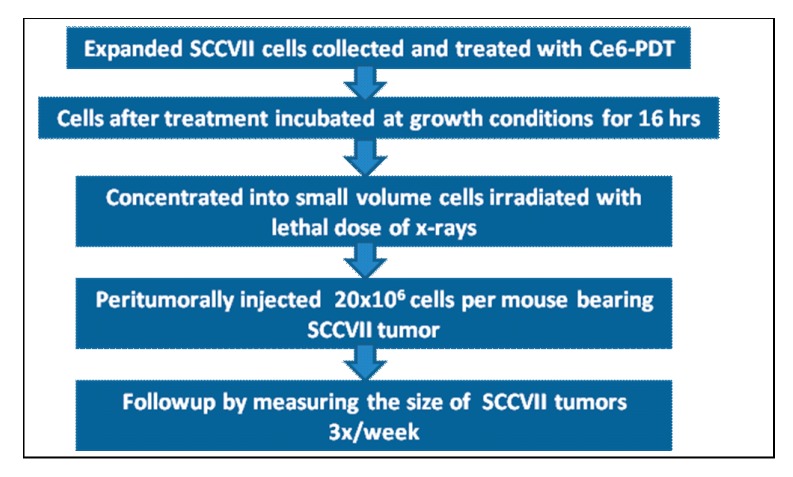
Schematic representation of PDT vaccine preparation.

**Figure 9:**
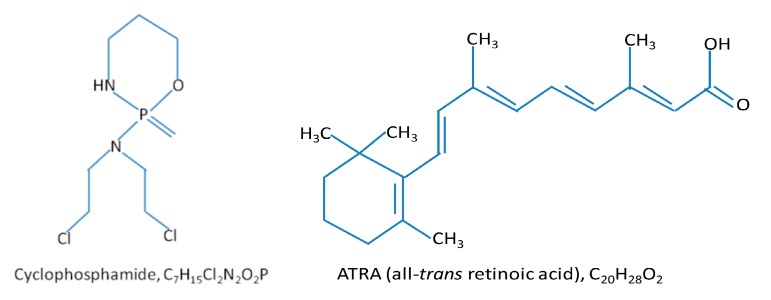
Chemical structures of cyclophosphamide and ATRA.

The protocol for vaccine preparation was described earlier (Figure 8) [26]. Briefly, after exposing to chlorin e6 (ce6) (Frontier Scientific Inc., Logan, UT, USA) at 0.5 µg/mL in serum-free medium for 30 min, SCCVII cells were treated by 1 J/cm^2^ of 665 ± 10 nm light. The light was produced by a 150 W quartz tungsten halogen (QTH) lamp high throughput illuminator model FB-QTH-3 (Sciencetech Inc., London, ON, Canada) with interchangeable interference filter with liquid light guide (model 77638, Oriel Instruments, Stratford, CT, USA) used for its delivery. The cells were left in EX-CELL chemically defined protein- and serum-free medium (Sigma Chemical Co., St. Louis, MO, USA) for 16 h at 37 °C, then collected, exposed to X-rays (60 Gy), and delivered (2 × 10^7^ cells/mouse) by peritumoral injection. Subsequent tumor size changes in vaccinated mice were monitored for assessing the therapeutic outcome. Some vaccinated and control mice received i.p. injection of cyclophosphamide (Sigma C0768) at 25 or 50 mg/kg, or all-*trans* retinoic acid (ATRA, also known as tretinoin, Sigma R2625) 40 mg/kg (Figure 9). The initial solvent for ATRA was chremophor-absolute ethanol (9:1); this was before injection diluted with saline (1:3) to 4 mg/mL. The sample size (6 animals) in these experiments was established based on power calculation for detecting differences in tumor response with an acceptable hazard ratio.

### 3.3. Flow Cytometry

For determining Treg cells, single cell suspensions were obtained from tumor tissues using a standard enzymatic digestion procedure [27]. Cells were then subjected to surface staining with anti-mouse CD4 and anti-mouse CD25 antibodies (both raised in rat) that were conjugated with fluorescein isothiocyanite (FITC) and phycoerythrin-cyanine5, respectively. This was followed by intracellular staining with phycoerythrin-conjugated rat anti-mouse Foxp3 antibody. All antibodies were from eBioscience Inc. (San Diego, CA, USA). To identify MDSCs, splenocytes were stained with rat anti-mouse GR1 conjugated with phycoerythrin-cyanine5 (eBioscience) and phycoerythrin-conjugated CD11b antibody produced in mouse (Santa Cruz Biotechnology Inc., Dallas, TX, USA). For flow cytometry, a Coulter Epics Elite ESP (Coulter Electronics, Hialeah, FL, USA) was used including 2 × 10^4^ cells for each test.

### 3.4. Foxp3 Gene Expression Analysis

Details of gene expression analysis were described previously [28,29]. Total RNA was extracted from mouse spleen tissue using Trizol and cleaned with Qiagen Min Elute (Qiagen Canada Inc., Montreal, QC, Canada). It was then used to create complementary strand DNA transcript that was amplified by quantitative RT-PCR in the presence of primers designed and tested in our laboratory that were specific for mouse Foxp3 gene (NCBI Reference Sequence NM_001199347). The structure of primers was GGCAGAGAGGTATTGAGGGTG (forward) and CTTTCTTCTGTCTGGAGTGGCT (reversed). The expression of housekeeping gene, glyceraldehide-3-phosphate dehydrogenase (GAPDH), was also determined and used for normalizing Foxp3 gene expression.

### 3.5. Statistical Analysis

Each of the experiments was repeated at least once. For statistical evaluation, the log-rank test was used for survival-type tumor response and Mann–Whitney test served for the analysis of other data; the threshold for statistical significance was set at 5%.
